# 975. A Custom, 3D Printed Textured Insole Improved Lower Extremity Function Following Grafting After Necrotizing Fasciitis

**DOI:** 10.1093/jbcr/irag033.572

**Published:** 2026-04-06

**Authors:** Valérie Calva, Zoë Edger-Lacoursière, Danielle Shashoua, Gregory Payet, Stéphanie Jean, Elisabeth Marois-Pagé, Bernadette Nedelec

**Affiliations:** Villa Medica Rehabilitation Hospital, Montréal, QC; Villa Medica Rehabilitation Hospital and McGill University, Montréal, QC; Villa Medica Rehabilitation Hospital, Montréal, QC; Villa Medica Rehabilitation Hospital, Montréal, QC; Université de Montréal, Montréal, QC; Villa Medica Rehabilitation Hospital, Montréal, QC; McGill University, Montréal, QC

## Abstract

**Introduction:**

Survivors of necrotizing fasciitis (NF) commonly undergo extensive debridement and skin grafting, which can cause significant functional limitations, especially when the foot is affected. These include difficulties walking, postural instability or challenges with complex work or leisure activities due to reduced sensation, proprioception, and increased pain. Textured insoles (TI) have shown promise in improving balance in stroke and diabetes populations. Thus, a 3D printed TI may be an innovative way to improve lower extremity (LE) function post NF. This report presents the pre-post measures of walking endurance, balance, patient-perceived LE function, edema, and sensory perception of a patient who underwent skin grafting after NF treated with a custom 3D-printed TI.

**Methods:**

A very active 51-year-old with idiopathic lymphedema underwent debridement and grafting of the dorsum of his foot and anterior lower leg due to NF. Eleven months post-surgery, he experienced persistent LE dysfunction and sensory deficits, which limited his walking endurance and ability to perform complex activities such as running, jumping, and squatting. A TI was designed using computer-aided (CAD) software by modifying an open-source insole file with added surface cylinders to stimulate plantar sensation during weight bearing. A 3D-printed TI using TPU at 30% infill was generated, then a closed-cell foam arch support was glued on to reduce foot pronation. The insole wearing time was gradually increased from 30 minutes to >7 hours a day across 6 weeks. Walking endurance and balance were assessed without and with the insole using the 6 Minute Walk Test (6MWT) and the mini-BESTest. Prior to wearing the insole and 4 weeks after wearing it for >7 hrs/day LE function, edema, and sensory perception were measured using the Lower Extremities Functional Scale (LEFS), the foot figure-of-eight, and Semmes Weinstein Monofilaments (SWM) respectively.

**Results:**

The patient’s 6MWT improved by 46 meters with the insole. The mini-BESTest increased from 26/28 to 28/28, with the patient being able to perform more complex tasks. The LEFS improved by 14 points 4 weeks post insole introduction. Foot edema decreased by 1.6 cm. Touch perception of the plantar foot evaluated with SWM improved from 6 g to 2 g.

**Conclusions:**

A custom, 3D-printed TI improved LE impairment, function, and facilitated participation in important leisure activities.

**Applicability of Research to Practice:**

This is the first documented case of functional improvements using a custom 3D-printed TI in a NF patient. It may also benefit burn survivors or others with LE sensory deficits.

**Funding for the study:**

N/A.

